# Is Chest Compression Superimposed with Sustained Inflation during Cardiopulmonary Resuscitation an Alternative to 3:1 Compression to Ventilation Ratio in Newborn Infants?

**DOI:** 10.3390/children8020097

**Published:** 2021-02-02

**Authors:** Seung Yeon Kim, Gyu-Hong Shim, Georg M. Schmölzer

**Affiliations:** 1 Centre for the Studies of Asphyxia and Resuscitation, Neonatal Research Unit, Royal Alexandra Hospital, Edmonton, AB T5H 3V9, Canada; dunggiduk@eulji.ac.kr (S.Y.K.); peddoc@paik.ac.kr (G.-H.S.); 2 Department of Pediatrics, Eulji University Hospital, Daejeon 35233, Korea; 3 Department of Pediatrics, Inje University Sanggye Paik Hospital, Seoul 01757, Korea; 4 Department of Pediatrics, Faculty of Medicine and Dentistry, University of Alberta, Edmonton, AB T6G 2R3, Canada; 5 Division of Neonatology, Department of Pediatrics and Adolescent Medicine, Medical University of Graz, Graz 8036, Austria

**Keywords:** newborn, neonatal resuscitation, chest compressions, sustained inflation

## Abstract

Approximately 0.1% for term and 10–15% of preterm infants receive chest compression (CC) in the delivery room, with high incidence of mortality and neurologic impairment. The poor prognosis associated with receiving CC in the delivery room has raised concerns as to whether specifically-tailored cardiopulmonary resuscitation methods are needed. The current neonatal resuscitation guidelines recommend a 3:1 compression:ventilation ratio; however, the most effective approach to deliver chest compression is unknown. We recently demonstrated that providing continuous chest compression superimposed with a high distending pressure or sustained inflation significantly reduced time to return of spontaneous circulation and mortality while improving respiratory and cardiovascular parameters in asphyxiated piglet and newborn infants. This review summarizes the current available evidence of continuous chest compression superimposed with a sustained inflation.

## 1. Introduction

Approximately 0.1% of term infants and 10–15% of preterm infants receive chest compressions (CC) in the delivery room (DR) [1,2,3,4,5]. Infants who receive CC have a high incidence of mortality and neurodevelopmental impairment [1,2,3,4,5]. Furthermore, newborns who received prolonged CC and epinephrine without signs of life at 10 min after birth have 83% mortality, with 93% of survivors suffering moderate-to-severe neurological disability [6,7]. The poor prognosis associated with receiving CC in the DR has raised concerns as to whether specifically-tailored cardiopulmonary resuscitation (CPR) methods could improve outcomes.

In newborn infants, bradycardia or cardiac arrest is mainly caused by hypoxia rather than a primary cardiac disease [8,9]. Therefore, the neonatal resuscitation guidelines put an emphasis on ventilation and adequate oxygen delivery. Current neonatal resuscitation guidelines recommend initiating CC if an infant’s heart rate remains < 60 beats/min, despite adequate ventilation for at least 30 s [8,9]. CC should be delivered at a rate of 90/min in sequences of three CC followed by a pause to deliver 1 inflation at a rate of 30/min, which corresponds to a 3:1 compression:ventilation (C:V) ratio [8,9]. The 3:1 C:V ratio is recommended, as respiratory failure is the primary cause of bradycardia or asystole in newborn infants [8,9]. A 3:1 C:V ratio has a higher rate of inflations compared to the pediatric or adult C:V ratios, which will result in a higher oxygen delivery, hence improved ventilation [8,9]. While the current neonatal resuscitation guidelines recommend a 3:1 C:V ratio, the most effective C:V ratio in newborn infants remains controversial.

Several studies have compared various C:V ratios or continuous chest compression with asynchronized ventilation [10,11,12,13,14], however none of the studies reported any improved outcomes compared to the 3:1 C:V ratio. More recently, our group used a higher airway pressure or sustained inflation during continuous chest compression (CC + SI), which significantly improved time to return of spontaneous circulation (ROSC) and survival [15]. While the current available data is mostly limited to animal data, some human data are available. The aim of the review is to provide an in-depth analysis of CC + SI during neonatal CPR.

## 2. 3:1 Compression-to-Ventilation Ratio: Rationale and Evidence

Current resuscitation guidelines in newborns recommend a 3:1 C:V ratio [8,9], however this approach may not be optimizing coronary and cerebral perfusion while providing adequate ventilation to improve outcomes. Animal studies on cardiac arrest demonstrated that combining CC with ventilations, compared with ventilations or CC alone, improves ROSC and neurological outcome at 24 h in asphyxiated newborn piglets [16,17,18].

Solevåg et al. compared 9:3 C:V and 15:2 C:V to 3:1 C:V in asphyxiated newborn piglets with cardiac arrest and reported no significant differences in the time to ROSC [10,11]. These studies suggest that just using a higher C:V ratio does not improve outcome in asphyxiated newborn piglets. Alternatively, continuous CC with asynchronous ventilations (CCaV), where 90 CC are given continuously with 30 non-synchronized inflations, would potentially improve hemodynamics during CC as there are no interruptions. Indeed, a manikin study reported a significantly higher minute ventilation with CCaV compared to 3:1 C:V ratio (221 vs. 191 mL/kg/min, respectively) [19]. During CPR in asphyxiated newborn piglets, CCaV or 3:1 C:V had similar minute ventilation (387 vs. 275 mL/kg) and similar time to ROSC (114 and 143 s for CCaV and 3:1 C:V, respectively) and survival (3/8 and 6/8, respectively) between the two groups [12,13]. Furthermore, no differences in diastolic blood pressure or mean arterial blood pressure between CCaV and 3:1 C:V were observed [12,13]. These studies suggest that CCaV has no advantage compared to 3:1 C:V.

## 3. Chest Compression with Sustained Inflations (CC + SI)

Schmölzer et al. compared continuous CC superimposed with a high distending pressure (or sustained inflation = CC + SI) with 3:1 CV during CPR of asphyxiated newborn piglets and reported (i) significantly reduced time to ROSC (median (Interquartile range (IQR)) 38 (23–44) vs. 143 (84–303) s, respectively, (*p* = 0.0008), mortality (7/8 (87.5%) vs. 3/8 (37.5%), respectively, *p* = 0.038), epinephrine administration (0/8 vs. 7/8, respectively, *p* < 0.0001), and improved systemic and regional hemodynamic recovery; (ii) less infants received 100% oxygen (3/8 vs. 8/8, respectively, (*p* = 0.0042); (iii) minute ventilation (mean (SD) 936 (201) vs. 623 (116) mL/kg/min, respectively, *p* = 0.0080), and therefore alveolar oxygen delivery, was significantly increased with CC + SI; iv) compression of the chest during SI forced gas out of the chest and during passive chest recoil allowed air to be drawn back into the lungs [15]. 

During CC + SI, CCs are delivered continuously and superimposed by a constant high airway pressure or sustained inflation (SI). During CC + SI, a constant high airway pressure or SI is given for a set time (e.g., 30 s) with a set peak inflation pressure (e.g., 25 cm H_2_O) while CCs are continuously delivered [20,21,22,23,24]. During compression and release phase, the distending pressure is fluctuating by ~1 cm H_2_O. After the set time (i.e., 30 s), the SI is paused for 1 s while CCs are continued. The SI is then resumed for the same time frame (i.e., 30 s). Both CC and SI combined as CC + SI are continued until ROSC. While in all studies a 1 s pause between each SI was used, the optimal duration for the pause between each SI (e.g., 0.5, 1, 2 s) has never been examined.

### Mechanism of CC + SI

Antegrade blood flow during CPR can be achieved by either direct cardiac compression between the sternum and vertebral column or increased intrathoracic pressure produced by CC [25]. Indeed, maneuvers that increase the intrathoracic pressure result in increased carotid blood flow during CPR, further augmenting antegrade blood flow [26,27]. Chandra et al. combined ventilation at high airway pressure while simultaneously performing CC in an animal model and demonstrated increased carotid flow, without compromising oxygenation [26,27]. Furthermore, providing continuous CC and lung inflation simultaneously substantially improved brain perfusion by enhancing cerebral perfusion pressure in a piglet model [28,29]. In addition, animal studies have demonstrated that an SI also increases intrathoracic pressure without impeding blood flow [30]. These data suggest that CC + SI might provide two maneuvers, which increase intrathoracic pressure and thereby improve blood flow. 

## 4. Chest Compression Rate

The newborn infant normal respiratory rate and resting heart rate are 40–60 breaths/min and 120–160/min, respectively. In comparison, the current neonatal resuscitation guidelines recommend CC with 90 compressions and 30 inflations per minute [8,9], which is lower than the normal physiological parameters.

Schmölzer et al. compared CC + SI with a CC rate of 120/min with 3:1 C:V with a CC rate of 90/min in a newborn asphyxiated piglets experiment, and reported shorter time to ROSC (38 (23–44) vs. 143 (84–303) s; (*p* = 0.0008)) and survival to 4 h with 7/8 vs. 3/8, respectively [15]. Similarly, Vali et al. reported that CC + SI with CC rate of 120/min was as effective as CC with 90/min with a 3:1 C:V ratio in achieving ROSC [31]. Li et al. compared CC rates of 90/min and 120/min during CC + SI and reported a reduced time to ROSC (34 (28–156) vs. 99 (31–255) s *p* = 0.29), respectively [32]. Those studies suggest the 90/min CC rates in the CC + SI might be sufficient to deliver an adequate tidal volume and minute ventilation without impairing gas exchange. However, a mathematical model suggests that the most effective CC rate depends on body size and body weight, and CC rates of 180/min for term infants and even higher for preterm infants might improve survival [33]. The mathematical model calculated that the optimal systemic perfusion pressure occurs at CC rates of 180 and 250/min for infants weighing 3 and 1 kg, respectively [33]. In infants and newborns, there are fundamental physical and mathematical reasons including (i) effects of the mass of venous blood columns entering the chest pump, (ii) length, and (iii) area scale with body size [33]. However, these higher CC rates might be impossible during manual CPR as healthcare professionals will get fatigued more quickly, which conversely affects CC quality [34,35,36]. Using an automated CC machine might be the solution to achieving these high CC rates. While automated CC machines are routinely used in adults, no such device is currently available for newborn infants.

## 5. Peak Inflation Pressures

The optimal peak inflation pressure during CC + SI for adequate tidal volume delivery is unknown. While the current neonatal resuscitation guidelines recommend an initial distending pressure of 20–25 cm H_2_O during positive pressure ventilation [8,9], the optimal peak inflation pressure remains unknown. During mask ventilation, a certain threshold peak inflation pressure is needed to move the liquid air interface downwards towards the alveoli [37,38]. Similarly, during CC + SI, a threshold sustained pressure is needed to deliver an adequate tidal volume. Solevåg et al. used manikins and cadaver piglets to establish the distending pressure required to achieve sufficient tidal volume delivery during CC + SI [39]. A distending pressure of 25 cm H_2_O was required to achieve a tidal volume delivery of > 5 mL/kg [39]. Tidal volume increased with increasing distending pressure in all models, with an overall positive correlation (*r* = 0.49, *p* < 0.001) [39]. Shim et al. compared a peak inflation pressure of 10, 20, and 30 cm H_2_O during CC + SI in asphyxiated newborn piglets and reported no difference in median (IQR) time to ROSC, with 75 (63–193), 94 (78–210), and 85 (70–90) s, respectively (*p* = 0.56) [22]. In addition, tidal volume was positively correlated with increasing pressure with a mean (SD) 7.3 (3.3), 10.3 (3.1), and 14.0 (3.3) mL/kg;(*p* = 0.0018) with 10, 20, and 30 cm H_2_O, respectively [22]. The higher tidal volume with a peak inflation pressure of 30 cm H_2_O also showed increased concentrations of proinflammatory cytokines interleukin-1β and tumor necrosis factor-α in the frontoparietal cerebral cortex (both *p* < 0.05 vs. sham-operated controls). These data suggest that pressures of 20–25 cm H_2_O might be sufficient to deliver an adequate tidal volume during CC + SI, and that higher pressures could lead to increases in lung inflammation markers.

## 6. Passive Ventilation

Tsui et al. applied a downward force of 0.16 kg per kg patient weight on the chest of infants undergoing surgery during general anesthesia and was able to deliver a tidal volume of 2.4 mL/kg or ~33% of an infant’s physiological tidal volume [40]. This study suggests that chest recoil produces a distending pressure-dependent tidal volume, which achieves passive ventilation during CCs. In asphyxiated term piglets, the delivered tidal volume was 10–15 mL/kg with a constant distending pressure of 25–30 cm H_2_O, and in preterm infants < 32 weeks’ gestation, the tidal volume ranged between 0.6 to 4.4 mL/kg with a constant distending pressure of 24 cm H_2_O [15,41]. These data demonstrate that passive ventilation is achieved when providing a constant high distending pressure during CC.

## 7. Tidal Volume

Providing adequate ventilation is a cornerstone of neonatal CPR. The main purpose of lung inflations during CCs is to provide an adequate tidal volume to facilitate oxygen delivery and gas exchange. However, during CPR with 3:1 C:V, Li et al. reported a cumulative loss of expiratory tidal volume of 4.5 mL/kg with each 3:1 C:V cycle [42], which could cause lung derecruitment and thereby interfere with oxygenation and ROSC. In comparison, during CC + SI, a constant lung recruitment and thereby gain in functional residual capacity was observed with a tidal volume gain of 2.4 mL/kg per CC + SI cycle [42]. This is supported by data from a human pilot trial comparing CC + SI with 3:1 C:V in the DR using a distending pressure of 24 cm H_2_O (local hospital policy during neonatal resuscitation) in preterm infants < 32 weeks of gestation [41,43]. During CC + SI, a significantly higher tidal volume and minute ventilation was delivered, suggesting that CC + SI might improve ventilation and oxygenation during neonatal CPR. During CC + SI, adequate tidal volume delivery might lead to better alveolar oxygen delivery and lung aeration, hence faster ROSC compared to 3:1 C:V group.

## 8. Duration of Sustained Inflations

SI as initial respiratory support in the DR has been postulated to achieve a more unified lung aeration [44]. However, recent systematic reviews reported similar rates of bronchopulmonary dysplasia when SI was compared with intermittent positive pressure ventilation for initial respiratory support in the DR [45,46]. These reviews also reported that in a subgroup of <28 weeks’ gestation, SI was associated with potential increased risk of death before discharge (risk ratio 2.42 (95% confidence interval = 1.15–5.09)) and increased risk of death within the first 2 days (risk ratio 1.38 (95% confidence interval = 1.00–1.91)), when compared to intermittent positive pressure ventilation [45,46]. However, the mechanism of how an initial SI could potentially increase risk of death is unknown.

Furthermore, the European resuscitation guidelines recommend five SIs of 3 s in asphyxiated term infants [47], though no human studies have examined this approach in newborn infants. However, a recent study in asphyxiated lambs reported that a 30 s SI will achieve lung aeration and hemodynamic stability, while five SIs of 3 s does not [48]. In the original study, we used a 30 s SI during CC + SI, which significantly reduces time to ROSC compared to 3:1 C:V ratio [15]. However, the optimal duration of SI to improve ROSC and reduce mortality during CC + SI remains unknown. Mustofa et al. compared CC + SI with either 20 s or 60 s in asphyxiated piglets and reported similar time to ROSC and survival, with no difference in tidal volume delivery [21]. In addition, there were no differences in markers of lung inflammation (IL-1ß, IL-6, IL-8, and TNF-α) and brain inflammation (IL-1ß, IL-6, and IL-8) between the groups [21]. This suggests that the duration of SI during CC + SI might be not the dependent factor, however further studies are needed to identify the optimal duration of SI during CC + SI.

## 9. Oxygen Concentration with CC + SI

The current neonatal resuscitation guidelines recommend 100% oxygen once CCs are initiated [8,9]. However, this is based on expert opinions and not supported by any clinical data. Several animal studies compared 21% or 100% oxygen during CC using the 3:1 C:V ratio in asphyxiated newborn piglets and reported no difference in time to ROSC or mortality. In addition, the cumulative alveolar oxygen exposure during resuscitation was significantly lower in the CC + SI group compared to the 3:1 C:V group, with mean (SD) 27,755 (4706) and 47,729 (6692) mmHg seconds, respectively (*p* < 0.001). Similar, a meta-analysis of these animal studies reported no difference in time to ROSC (mean difference of −3.8 (−29.7–22) s, I^2^ = 0%, *p* = 0.77) or mortality (risk ratio 1.04 (0.35, 3.08), I^2^ = 0%, *p* = 0.94) between 21% or 100% oxygen during CC with the 3:1 C:V ratio [49]. Recently, Hidalgo et al. compared 21% and 100% oxygen during CC + SI in term newborn asphyxiated piglets and reported similar time to ROSC (median (IQR) 80 (70–190) vs. 90 (70–324) s, respectively, *p* = 0.56), short-term survival (7/8 (88%) vs. 5/8 (63%), respectively, *p* = 0.569), and hemodynamic recovery [50]. In addition, there was no significant difference in injury markers in the left ventricle tissue or the frontoparietal cortex tissue. These data suggest that 21% oxygen during CPR might be efficient, however human data are needed.

## 10. Type of Cardiac Arrest

In 2015, the neonatal resuscitation guidelines added the use of an electrocardiograph to assess heart rate at birth [51,52]. This led to several reports of pulseless electrical activity during CPR in the DR [53,54]. In addition, rates of up to 50% of asphyxiated piglets displayed pulseless electrical activity during asphyxia-induced cardiac arrest [55,56,57]. Solevåg et al. reported that cardiac arrest due to pulseless electrical activity will result in lower rates of ROSC and lower 4 h survival, compared to asystole, in asphyxiated newborn piglets [56]. This suggests that the initial electrocardiograph algorithm might serve as an outcome predictor during neonatal CPR.

## 11. Inflammatory Markers

There are concerns that SI could adversely affect lung or brain injury. Lista et al. reported a pneumothorax rate of 6% compared to 1% with intermittent positive pressure in preterm infants with 25–28 weeks of gestation [58]. However, the mechanisms for an increased rate of pneumothorax during SI is unknown. Interestingly, none of the animal studies examining CC + SI reported pneumothoraxes during autopsy. There is also the concern that SI delivers an excessive large tidal volume, which could cause a pulmonary proinflammatory response and initiate systemic inflammatory cascade [59]. However, when SIs were given as initial respiratory support, no increase in lung injury marker has been reported [60,61]. Similar, during CPR with either CC + SI or 3:1 C:V, no difference in lung injury markers were observed.

The mechanism of brain injury is thought to be impaired venous return or secondary brain injury due to excessive tidal volume delivery. Sobotka et al. reported that a single 30 s SI followed by ventilation caused a blood–brain barrier disruption and cerebral vascular leakage, which may exacerbate brain injury in asphyxiated near-term lambs [62]. This injury might have occurred as a direct insult of the initial SI or due to the excessive tidal volume delivered during subsequent ventilation. Recently, Shim et al. reported that a peak inflation pressure of 30 cm H_2_O delivered a significant higher tidal volume compared to peak inflation pressure of 20 cm H_2_O, which was associated with significant increased cerebral tissue pro-inflammatory cytokines [22]. While CC + SI did not increase lung injury markers, markers of brain inflammation were increased, and therefore a peak inflation pressure of ≥ 25 cm H_2_O should not be exceeded. 

## 12. Clinical Studies

The animal data suggest that CC + SI might be an effective CC technique for newborn infants. A pilot trial compared CC + SI (*n* = 5) with 3:1 C:V (*n* = 4) in preterm infants < 32 weeks’ gestation with a mean (SD) gestational age of 24.6 (1.3) and 25.6 (2.3) weeks [41]. There was a significantly shorter time to ROSC with CC + SI, compared to 3:1 C:V, with 31(9) vs. 138 (72) s, respectively (*p* = 0.011) [41]. In addition, CC + SI provided a higher minute ventilation and ventilation rate, while short-term outcomes, including intraventricular hemorrhages, air leak, retinopathy of prematurity, and chronic lung disease, were similar between groups [41]. Although mortality was higher in the CC + SI group with 2/5 vs. 0/4 in the 3:1 C:V group, this did not reach statistical significance, as the sample size was too small, and it was a very vulnerable patient population.

Currently, the Sustained Inflation and Chest Compression Versus 3:1 Chest Compression to Ventilation Ratio During Cardiopulmonary Resuscitation of Asphyxiated Newborns: A Randomized Controlled Trial (SURV1VE-trial) is recruiting term and preterm infants born > 28^+0^ weeks’ gestational age requiring chest compression in the delivery room [63,64]. In this cluster trial, hospitals are randomized to either CC + SI or 3:1 C:V ratio for one year each [63,64]. The SURV1VE-trial has been approved by a human clinical research ethical committee at all participating sites, and a Data Safety Monitoring Committee is assessing the results of the trial at regular intervals to assure safety. The SURV1VE-trial hypothesis is that in newborn infants, CC + SI, compared to 3:1 C:V, during CPR will reduce the time needed to ROSC, and aims to recruit 218 participants (109 control group and 109 intervention group). The SURV1VE-trial aims to be completed by 2024.

## 13. Limitations

There are several limitations which prevent routine use of CC + SI in the DR. Most animal studies described in this review used piglets that have already undergone the fetal-to-neonatal transition. All experimental animals were sedated/anesthetized and intubated with a tightly sealed endotracheal tube to prevent any endotracheal tube leak, which may not occur in the delivery room as mask ventilation is frequently used [65].

Furthermore, sustained lung inflations have been postulated as a ventilation strategy immediately after birth [44]. Indeed, in intubated and sedated animals, SI improved lung aeration compared to intermittent positive pressure ventilation. However, several smaller randomized trials and meta-analyses were unable to identify any advantage or disadvantage for either SI or intermittent positive pressure ventilation [66]. Recently, the SAIL trial compared SI with intermittent positive pressure ventilation in < 28 weeks’ gestation infants and reported an increased mortality within the first 48 h with SI [67]. Most recently, a meta-analysis from ILCOR raised concerns about the potential harm of SI for premature infants < 28 weeks’ gestation [46]. These data raise some concerns about the use of SI during the initial respiratory support.

## 14. Conclusions

CC + SI reduces time to ROSC, improves mortality, and improves respiratory and hemodynamic parameters compared to 3:1 C:V ratio during neonatal CPR. CC + SI allows for passive lung ventilation and adequate tidal volume. Peak inflation pressures of 20–25 cm H_2_O might be sufficient to deliver an adequate tidal volume during CC + SI, and higher pressures could lead to increases in lung inflammation markers. Furthermore, 21% oxygen had similar time to ROSC or mortality compared to 100% oxygen. However, more clinical data are needed before this can be routinely used in the delivery room during neonatal chest compression.

## Data Availability

All data are presented within the article.

## Abbreviations

CC	chest compression
DR	delivery room
CPR	cardiopulmonary resuscitation
C:V	compression:ventilation
SI	sustained inflation
CC + SI	sustained inflation during chest compression
ROSC	return of spontaneous circulation
CCaV	continuous CC with asynchronous ventilations
